# Assessing causal association of circulating micronutrients and systemic lupus erythematosus susceptibility: a Mendelian randomization study

**DOI:** 10.3389/fnut.2024.1359697

**Published:** 2024-08-05

**Authors:** Shihui Huang, Xuemei Wei, Fang Qin, Zongxiang Yuan, Chuye Mo, Yiwen Kang, Chunlin Huang, Junjun Jiang, Li Ye

**Affiliations:** ^1^Guangxi Key Laboratory of AIDS Prevention and Treatment, School of Public Health, Guangxi Medical University, Nanning, China; ^2^Maternal and Child Health Hospital of Guangxi Zhuang Autonomous Region, Nanning, China; ^3^Collaborative Innovation Centre of Regenerative Medicine and Medical BioResource Development and Application Co-constructed by the Province and Ministry, Life Science Institute, Guangxi Medical University, Nanning, China

**Keywords:** circulating micronutrients, minerals, vitamins, systemic lupus erythematosus, Mendelian randomization

## Abstract

**Background:**

Previous studies showed the conflicting associations between circulating micronutrient levels and systemic lupus erythematosus (SLE). Therefore, we aimed to clarify the causal association between circulating micronutrient levels and the risk of SLE by two-sample Mendelian randomization (MR) analysis.

**Methods:**

56 single nucleotide polymorphisms (SNPs) significantly associated with 14 circulating micronutrients (vitamin A, B6, B9, B12, C, D and E, phosphorus, calcium, magnesium, copper, iron, zinc, and selenium) in published genome-wide association studies (GWAS) were used as instrumental variables (IVs). And summary statistics related to SLE were obtained from the IEU OpenGWAS database. We used the MR Steiger test to estimate the possible causal direction between circulating micronutrients and SLE. In the MR analysis, inverse variance weighting (IVW) method and the Wald ratio was as the main methods., Moreover, the MR-Pleiotropy residuals and outliers method (MR-PRESSO), Cochrane’s Q-test, MR-Egger intercept method and leave-one-out analyses were applied as sensitivity analyses. Additionally, we conducted a retrospective analysis involving the 20,045 participants from the Third National Health and Nutritional Examination Survey (NHANES III). Weight variables were provided in the NHANES data files. Univariate and multivariate logistic regression analyses were performed to determine the associations between circulating micronutrients and SLE.

**Results:**

The MR estimates obtained from the IVW method revealed potential negative correlations between circulating calcium (OR: 0.06, 95% CI: 0.01–0.49, *P* = 0.009), iron levels (OR: 0.63, 95% CI: 0.43–0.92, *P* = 0.016) and the risk of SLE. The results remained robust, even under various pairs of sensitivity analyses. Our retrospective analysis demonstrated that the levels of vitamin D, serum total calcium, and serum iron were significantly lower in SLE patients (*N* = 40) when compared to the control group (*N* = 20,005). Multivariate logistic regression analysis further established that increased levels of vitamin D and serum total calcium served as protective factors against SLE.

**Conclusion:**

Our results provided genetic evidence supporting the potential protective role of increasing circulating calcium in the risk of SLE. Maintaining adequate levels of calcium may help reduce the risk of SLE.

## 1 Introduction

Systemic lupus erythematosus (SLE) is a chronic, debilitating, multi-system autoimmune disease characterized by wide-ranging clinical manifestations (1), with high morbidity and mortality (2). The global prevalence and fatality rates of SLE have been documented as 13-7713.5/100,000 and 0.01–2.71/100,000, respectively (3). Recently, dietary interventions in preventing autoimmune diseases have garnered increasing interest among researchers. Circulating micronutrients primarily obtained through dietary intake can notably influence physiological functions in the conditions of both overabundance and deficiency. Despite extensive research, the circulating micronutrients associated with SLE remain only partially understood.

Micronutrients are typically nutrients that cannot be synthesized by the body and generally consist of water-soluble vitamins, fat-soluble vitamins, trace elements, and trace minerals. In the past two decades, many studies have indicated that circulating micronutrients play a significant role in developing immunoinflammatory diseases, but the findings are still confusing (4–10). In numerous studies of patients with SLE, vitamin D deficiency was more common compared to those without SLE (11–13). However, two prolonged follow-up studies showed that vitamin D supplementation during adolescence had no preventive effect on the development of SLE in adulthood and adult women (9, 14). As an essential trace element, iron has been reported to be involved in a diversity of biological processes. Nevertheless, there are still limited findings on the role of iron in the pathogenesis of SLE. It was observed from two recent studies that sufficient iron status was inversely associated with the risk of developing SLE (15, 16). In contrast, a case report conducted by Oh VM illustrated that iron supplementation resulted in the manifestation of SLE-like symptoms in a woman of childbearing age suffering from iron deficiency anemia (17). Given that most of these studies are observational studies and prone to confounding factors and reverse causation. Therefore, a more detailed elucidation of the potential causal relationship and causal direction between circulating micronutrients and SLE is urgently necessary.

Mendelian randomization (MR) leverages genetic variations associated with exposure as unconfounded instrumental variables (IVs) to evaluate the causal relationship between exposure and outcome (17). This method limits both bias and reverse causality, which commonly occurs in observational epidemiological studies (18). In theory, MR operates on a similar principle to naturally occurring randomized controlled trials (RCTs) and serves as a pivotal approach to strengthening causal inference in situations where conducting RCTs is impractical or unethical (19). Given the multiple advantages of MR in inferring the causal relationship between exposure and outcome, our study utilized a two-sample MR analysis to investigate potential causal relationships between genetically predicted 14 circulating micronutrients (including vitamins and minerals) and the risk of SLE.

## 2 Materials and methods

### 2.1 Study design

**FIGURE 1:**
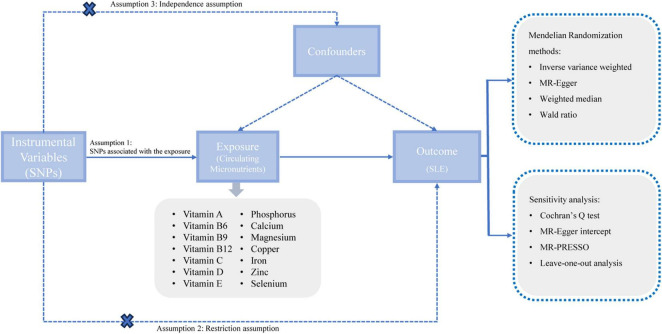
A schematic representation of our study design. SNPs, single nucleotide polymorphisms; SLE, systemic lupus erythematosus; MR, Mendelian randomization.

This study adhered to the guidelines stipulated by the Strengthening the Reporting of Observational Studies in Epidemiology using Mendelian randomization (STROBE-MR) (20). The STROBE-MR checklist for the reporting of MR studies was showed in Supplementary Table 1. We utilized the two-sample MR method to investigate the potential causal relationships between circulating micronutrients and the risk of SLE. Given that our study harnessed data extracted from pre-existing published literature, it circumvented the need for further ethical approval or informed consent. The architecture of our study was based on the three core assumptions underpinning MR (Figure 1).

### 2.2 Selection of genetic instrumental variables

A systematic search of PubMed was conducted to identify observational studies published on circulating micronutrients in relation to SLE. This resulted in an initial list of such micronutrients, comprising vitamin A, vitamin B6, vitamin B9, vitamin B12, vitamin C, vitamin D, vitamin E, sodium, phosphorus, calcium, magnesium, copper, iron, zinc, and selenium (4). Although several MR studies have evaluated the role of vitamin B9 (21), vitamin B12 (21), vitamin D (22), and iron status (16) in SLE, the Genome-Wide Association Study (GWAS) data for both exposures and outcomes used in our research exhibit slight variations. As a result, we have undertaken a replication of these analyses. Following this, we explored the GWAS catalog and PubMed for published GWAS centered on circulating micronutrients in European populations (the search was last updated in September 2023). Owing to the lack of relevant studies on sodium, it was excluded from our analysis. Ultimately, our research encompassed GWAS of 14 different circulating micronutrients: vitamin A (23), vitamin B6 (24), vitamin B9 (25), vitamin B12 (25), vitamin C (26), vitamin D (27), vitamin E (28), phosphorus (29), calcium (30), magnesium (31), copper (32), iron (33), zinc (32), and selenium (32). In this research, Single Nucleotide Polymorphisms (SNPs) linked to these 14 circulating micronutrients were designated as instrumental variables (IVs) adhering to the following standards: (1) The SNP demonstrates significant association with circulating micronutrient (*P* < 5e-08) and lacks linkage disequilibrium (*r*^2^ < 0.001, KB = 10,000) (34); (2) The SNP with a minor allele frequency (MAF) of ≥ 5% (35); (3) The SNP showing no evidence of reverse causality, as determined by the Steiger filtering test (36); (4) In cases where the SNP is not found in the results dataset, a closely associated SNP (*r*^2^ > 0.8) is chosen as a proxy in the 1000 Genomes database. If proxy SNP was unavailable, it was excluded from the analysis (37). (5) The chosen SNP is confirmed to be unassociated with confounding factors through inspection via the PhenoScanner database^1^ (*P* < 5e-08,*r*^2^ = 0.8) (38). Furthermore, we calculated the *R*^2^ to denote the variance explained by the SNP and the F-statistic to signify potential weak IV bias in MR analysis. The *R*^2^ was calculated as follows (39): *R*^2^ = 2 × Beta^2^ × (1-EAF) × EAF/SD^2^, and the F-statistic was calculated as (40): *F* = (Beta)^2^/(SE)^2^, where Beta is the per allele effect size of the association between each SNP and phenotype, EAF is the effect allele frequency, SE is the standard error, SD is the standard deviation. The IV is deemed strong when the F-statistic ≥ 10 (40). Ultimately, we identified 56 SNPs correlated with 14 circulating micronutrients, serving as IVs. The summary statistics of these SNPs utilized for MR analysis are presented in Table 1 and Supplementary Table 2.

**TABLE 1:** Circulating micronutrient-associated SNPs used as instrumental variables in the Mendelian randomization analyses.

Exposure	SNPs	EA	OA	EAF	Beta	SE	*P*-value	MR Steiger test (*P*-value)
**Vitamins**								
Vitamin A	rs10882272	C	T	0.35	−0.03	0.004	7.80E-12	9.65E-14
	rs1667255	C	A	0.31	0.03	0.004	6.35E-14	4.04E-13
Vitamin B6	rs4654748	C	T	0.50	−1.45	0.280	8.30E-18	3.17E-07
vitamin B9	rs1801133	G	A	0.67	0.11	0.008	6.65E-53	8.74E-43
	rs652197	C	T	0.18	0.07	0.010	5.73E-13	2.96E-10
	rs76630415	G	T	0.21	−0.04	0.007	2.40E-08	1.04E-06
Vitamin B12	rs1131603	C	T	0.06	0.19	0.017	4.30E-28	1.55E-25
	rs1141321	C	T	0.63	0.06	0.007	1.40E-16	2.17E-16
	rs12272669	A	G	0.00	0.51	0.086	3.00E-09	2.48E-08
	rs1801222	G	A	0.59	0.11	0.007	1.10E-52	2.37E-48
	rs2270655	G	C	0.94	0.07	0.016	3.50E-05	1.30E-04
	rs2336573	T	C	0.03	0.32	0.021	1.10E-51	1.69E-47
	rs34324219	C	A	0.88	0.21	0.012	8.80E-71	6.86E-58
	rs3742801	T	C	0.29	0.05	0.008	5.30E-08	1.73E-07
	rs41281112	C	T	0.95	0.17	0.016	9.60E-27	5.76E-23
	rs602662	A	G	0.60	0.16	0.008	4.10E-96	1.40E-79
Vitamin C	rs10051765	C	T	0.34	0.04	0.007	3.64E-09	2.95E-07
	rs10136000	A	G	0.28	0.04	0.007	1.33E-08	5.87E-08
	rs117885456	A	G	0.09	0.08	0.012	1.70E-11	1.12E-09
	rs13028225	T	C	0.86	0.10	0.009	2.38E-30	6.66E-27
	rs174547	C	T	0.33	0.04	0.007	3.84E-08	1.06E-05
	rs2559850	A	G	0.60	0.06	0.006	6.30E-20	6.69E-20
	rs56738967	C	G	0.32	0.04	0.007	7.62E-10	8.98E-06
	rs6693447	T	G	0.55	0.04	0.006	6.25E-10	1.56E-09
	rs9895661	T	C	0.82	0.06	0.008	1.05E-14	5.65E-13
Vitamin D	rs10741657	A	G	0.40	0.03	0.002	2.05E-46	4.88E-44
	rs10745742	T	C	0.40	0.02	0.002	1.88E-14	4.17E-15
	rs12785878	T	G	0.75	0.04	0.002	3.80E-62	6.05E-61
	rs17216707	T	C	0.79	0.03	0.003	8.14E-23	3.73E-15
	rs3755967	T	C	0.28	−0.09	0.002	1.00E-200	0.00E+00
	rs8018720	C	G	0.82	−0.02	0.003	4.72E-09	3.86E-07
Vitamin E	rs964184	G	C	0.21	0.04	0.010	7.80E-12	7.53E-05
**Minerals**								
Phosphorus	rs2970818	T	A	0.09	0.05	0.008	4.38E-09	8.73E-09
	rs9469578	C	T	0.92	0.06	0.009	1.11E-11	2.34E-10
	rs947583	T	C	0.29	0.04	0.005	3.45E-12	1.32E-10
Calcium	rs10491003	T	C	0.09	0.03	0.005	1.60E-06	2.05E-06
	rs1550532	C	G	0.31	0.02	0.003	4.60E-08	1.22E-08
	rs1570669	G	A	0.66	0.02	0.003	4.00E-08	1.07E-07
	rs7336933	G	A	0.15	0.02	0.004	1.60E-07	9.71E-08
	rs7481584	G	A	0.30	0.02	0.003	9.20E-10	1.39E-10
	rs780094	T	C	0.42	0.02	0.003	3.70E-11	9.17E-10
Magnesium	rs11144134	C	T	0.08	0.01	0.001	8.20E-15	5.76E-26
	rs13146355	A	G	0.44	0.01	0.001	6.30E-13	2.53E-06
	rs3925584	T	C	0.55	0.01	0.001	5.20E-16	1.23E-08
	rs4072037	T	C	0.54	0.01	0.001	2.00E-36	8.49E-22
	rs448378	A	G	0.53	0.00	0.001	1.25E-08	1.12E-04
	^a^rs7965584	A	G	0.71	0.01	0.001	1.10E-16	4.56E-11
Copper	rs1175550	G	A	0.23	0.20	0.032	5.03E-10	1.23E-09
	rs2769264	G	T	0.19	0.31	0.034	2.63E-20	1.14E-19
Iron	rs1800562	A	G	0.07	0.33	0.016	2.72E-97	1.89E-69
	rs7385804	T	G	0.67	−0.07	0.007	6.65E-20	7.52E-79
	rs8177240	T	G	0.67	−0.07	0.007	6.65E-20	8.91E-18
	rs855791	G	A	0.55	0.18	0.007	1.32E-139	3.21E-18
Zinc	rs1532423	A	G	0.43	0.18	0.026	9.00E-12	1.39E-11
	rs2120019	T	C	0.81	0.29	0.033	1.50E-18	1.35E-17
Selenium	rs921943	T	C	0.29	0.25	0.023	9.40E-28	1.05E-25

SNPs, single nucleotide polymorphisms; OA, other allele; EA, effect allele; SE, standard error. ^a^rs7965584 was not available in the outcome dataset, and rs11105470 was found to replace it in the 1000 Genomes database.

### 2.3 SLE data source

The GWAS summary data (GCST90018917) for SLE were sourced from a recent large-scale GWAS in the IEU OpenGWAS database. This dataset comprises 647 cases of European ancestry (from Finland and the UK) and 482,264 control subjects of European ancestry. Then, cases of non-European ancestry (from Japan) have been excluded.

### 2.4 Statistical analysis

Following the harmonization of SNPs in both exposure and outcome using identical alleles, a two-sample MR analysis was conducted. When the MR estimate contained only one single SNP, the Wald ratio method was adopted as the primary analysis method (41); when the number of SNPs was ≥ 2, we employed the inverse variance weighted (IVW) method as the primary analysis method (42). When the number of SNPs was ≥ 3, the MR-Egger and Weighted median methods were applied for supplementary approaches to test the robustness of the primary analysis (43, 44). Furthermore, an MR analysis was conducted separately for each SNP associated with exposures. In addition, to ensure that the MR effects were oriented in the correct direction (from exposure to SLE), we conducted the MR Steiger test to confirm that each instrumental variable (IV) explained more variance in the exposure than in the outcome (36).

The degree of heterogeneity amongst the IVs was evaluated using Cochrane’s Q test (45). When *P* < 0.05, it signifies the presence of heterogeneity. In cases of observed heterogeneity, the random effects IVW method is deployed to ascertain the causal relationship between exposure and outcome, thereby mitigating bias from heterogeneous IVs. The MR-Egger intercept detected horizontal pleiotropy in the IVs, with *P* < 0.05 indicating its presence (44). The MR-Pleiotropy Residual Sum and Outlier method (MR-PRESSO) was employed to identify outlying SNPs and rerun the analysis after outlier removal (46). Finally, the leave-one-out analysis was implemented to ascertain the MR analysis’s robustness and determine whether a specific SNP drove any association (47).

In this study, *P* < 0.05 was considered statistically significant. All analyses were performed using R software, with the “TwoSampleMR” and “MR-PRESSO” packages facilitating the two-sample MR analysis.

### 2.5 External validation in the NHANES III cohort

We utilized the NHANES III (1988–1994) data as the external validation dataset for this study. The NHANES III participants were restricted to adults aged 17 years and older. After excluding 5 participants with unknown SLE status, a total of 20,045 participants with the completed household interview and physical examination were included in the analysis. The participant’s SLE status was determined by the item in the questionnaire: “Doctor ever told you had: lupus?” The other variables including age, gender, and race were also derived from the household interview data, while BMI was calculated using the formula: BMI = weight (kg)/[height (m)]^2^. The serum levels of the 6 circulating micronutrients (vitamin A, vitamin C, vitamin D, serum calcium, iron, and selenium) were obtained from the laboratory examination data.

Considering the complex survey design, the weight variables were provided in the NHANES data files and *t*-tests, chi-square tests, and rank-sum tests were utilized to compare demographic disparities between the SLE group (*N* = 40) and the control group (*N* = 20,005). In the univariate regression analysis, we first constructed a preliminary rude model using only age, gender, race, and BMI. Then 6 circulating micronutrients were individually analyzed based on this rude model. Finally, variables with a significance level of *P* < 0.10 in above univariate regression analysis were included in the next multivariate regression analysis, which aimed to identify the most significant factors associated with SLE. And both the multivariate and univariate regression analyses were adjusted for the same covariates.

## 3 Results

### 3.1 The causal relationship of 14 circulating micronutrients on SLE in the European populations

**FIGURE 2:**
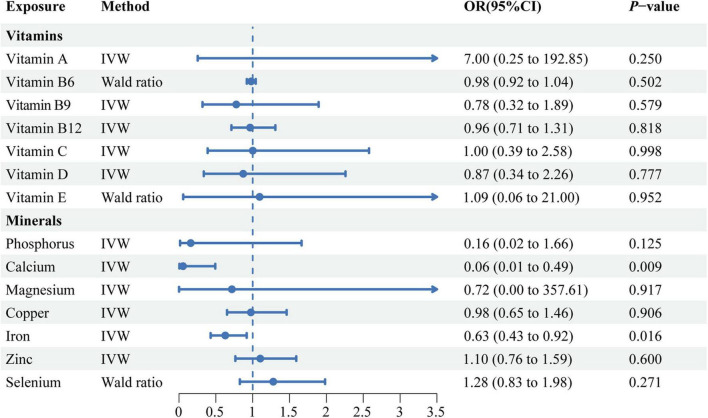
A forest plot showing the associations between genetically determined circulating micronutrients and systemic lupus erythematosus, based on Mendelian randomization analysis. IVW, inverse variance weighted; OR, odds ratio; CI, confidence interval.

The MR estimates obtained from the IVW method revealed potential negative correlations between circulating calcium (OR: 0.06, 95% CI: 0.01–0.49, *P* = 0.009), iron levels (OR: 0.63, 95% CI: 0.43–0.92, *P* = 0.016) and the risk of SLE (Figure 2). Concurrently, the weighted median method also derived similar results regarding the causal relationship between circulating iron level and the risk of SLE (OR: 0.60, 95% CI: 0.39–0.92, *P* = 0.020). The directional consistency of the causal relationship between circulating calcium level and the risk of SLE was maintained in the weighted median analysis (OR: 0.08, 95% CI: 0.00–1.18, *P* = 0.066) and IVW analysis, albeit without statistical significance (Supplementary Table 3). However, we did not observe significant correlations between vitamin A, vitamin B9, vitamin B12, vitamin C, vitamin D, vitamin E, phosphorus, magnesium, copper, zinc, selenium and the risk of SLE, as detailed in Supplementary Table 3.

### 3.2 Sensitive analysis

As indicated in Supplementary Table 4, both Cochrane’s Q test and the MR-Egger intercept suggest no heterogeneity and horizontal pleiotropy present in our MR analyses (*P* > 0.05). In the MR-PRESSO analyses, the rs1697421 (*P* = 0.01), rs17265703 (*P* = 0.005) and rs1801725 (*P* = 0.005) were identified as outliers. Then, no outlier SNPs were detected after removing and re-testing (*P* > 0.05) (Supplementary Table 4). When conducting the MR analyses using individual SNPs for either circulating calcium or iron levels, the results aligned with those obtained through the MR-PRESSO method (Supplementary Figure 1). The scatter plots, funnel plots, and leave-one-out plots all showed that the MR analysis results for the relationship between circulating calcium and iron levels with the risk of SLE remained robust, even under various pairs of sensitivity analyses (Supplementary Figures 2–4). Furthermore, as shown in Supplementary Table 5, the F-statistics for all 56 SNPs exceed 10, indicating no weak instrumental bias in our MR analyses.

### 3.3 Validation analysis in the NHANES III cohort

To further validate the findings in our MR analysis, we compared and analyzed the levels of circulating micronutrients in the serum of patients with SLE and those without SLE which sourced from a large cohort, known as the NHANES III cohort.

A total of 20,045 participants were ultimately included in this study. The demographic characteristics of the NHANES III participants by SLE status are presented in Supplementary Table 6. After applying appropriate weighting for the analysis, we observed that the mean age of the SLE group [52.06 (13.72)] was significantly higher than that of the control group [57.80 (13.92)] (*P* < 0.01). However, there were no significant differences observed in other demographic characteristics, including BMI, gender, and race. In the comparative analysis of 6 circulating micronutrients between the two groups, the SLE group exhibited significantly lower levels of vitamin D (*P* < 0.01), serum total calcium (*P* = 0.01), and serum iron levels (*P* = 0.04) (Supplementary Table 7). Consistent with the results of univariate Logistic regression analyses, multivariate Logistic regression analyses also found that vitamin D (OR: 0.98, 95% CI: 0.97–1.00, *P* = 0.01) and serum total calcium (OR: 0.03, 95% CI: 0.00–0.58, *P* = 0.02) had a protective effect against SLE (Supplementary Table 8).

## 4 Discussion

The precise etiology of SLE remains unclear. Recently, the potential of dietary interventions in preventing autoimmune diseases has garnered increasing interest among researchers. While there have been prior causal analyses involving single exposure, such as vitamin D, vitamin B, and iron status with SLE, to the best of our knowledge, this is the first comprehensive study to explore the causal associations between multiple circulating micronutrients and SLE. Our MR analyses showed the causal association between genetically predicted reductions in circulating calcium, iron and susceptibility to SLE in European populations. However, in the external validation analysis using the NHANES III cohort, only circulating calcium emerged as a protective factor for SLE.

In the present study, for the first time, we support a causal association between circulating calcium and SLE, and circulating calcium can serve as a potential protective factor against SLE. An earlier observational case-control study supported our results by finding a correlation between serum total calcium levels and activity of SLE (48). The researchers also observed that the serum calcium levels in SLE patients were significantly lower than those of healthy individuals (48). A retrospective analysis likewise discovered a significant reduction in the serum calcium levels of SLE patients when compared to those of healthy controls (49). Furthermore, a significant proportion of patients with SLE, as identified by numerous cross-sectional studies, exhibit insufficient levels of calcium intake, seldom reaching the recommended dietary allowance (15, 50). Calcium is an essential trace metal required for biological growth, and calcium signaling regulates many immune tolerance and inflammation pathways. Studies have found that the disruption of B-cell tolerance is a core key to the onset of SLE, and calcium signaling plays an important role in the development and fate of B cells (two key aspects of immune tolerance) through specific activation of transcription programs (51). Additionally, calcium signaling transmission can regulate the activation of the cGAS-STING axis, thus participating in innate immunity and autoimmune regulation through Type I interferon (52). Calcium exists in the blood in three forms (the ionic form, the form bound primarily to albumin, and the form bound to anions), with Ca^2+^ being the physiologically active form of calcium. Nonetheless, contemporary clinical laboratory routines continue to measure overall serum calcium levels to represent the calcium status of the body. Thus, serum calcium may serve as a potential biomarker for the onset and progression of SLE.

In the MR analysis, an elevation in serum iron levels is associated with a decreased risk of SLE, the findings congruent with those derived from recent MR investigations (16). However, our validation analysis in the NHANES III cohort revealed that, after adjusting for demographic characteristics, there was no significant association between serum iron and SLE, as indicated by the univariate analysis using Logistic regression. In fact, the association between serum iron and SLE is not clear, with inconsistent conclusions reported. A recent substantial cohort study conducted in China revealed that the risk of developing SLE is notably higher in patients with iron deficiency anemia (53). Another case-control study conducted in Bangladesh also revealed similar results (54). However, in two additional small-scale case-control studies, no substantial difference was observed in the serum iron levels between patients with SLE and their control counterparts (55, 56). There are also indeed conflicting research findings regarding the association between serum iron and the mechanisms of inflammation induction. Prior studies have established that iron serves as a crucial micronutrient required for the proliferation of B cells and the production of antigen-antibodies (57). Iron homeostasis is critical in the incidence and progression of autoimmune inflammatory diseases (58). Research has demonstrated a substantial correlation between iron homeostasis and immune inflammation. Iron deficiency could potentially influence the expression of cytokines such as IL-6, IL-1, TNF-α, and IFN-γ, contributing to tissue damage (59). However, Wang et al. discovered that an overabundance of iron could stimulate the generation of pro-inflammatory cytokines via poly(rC)-binding protein 1 (Pcbp1), consequently leading to the direct induction of autoimmune diseases (60). Therefore, further investigation through large-scale experimental epidemiological studies is needed to explore the association between serum iron and SLE.

The strengths of this study are as follows: First, we built the causal relationship between multiple circulating micronutrients and the risk of SLE in European populations and validate in the NHANES III cohort. This comprehensive analysis can provide a more global understanding of them. Second, the exposure and outcome of our study come from different regions of the same lineage, the overlap of samples is relatively light, and the bias of population stratification is small. Finally, we excluded SNPs that may have a reverse causality and overcame the limitations of observational studies (confounding factors, recall bias).

There are also some limitations in this study. First, although multiple MR methods were used to prevent confounding caused by pleiotropy, residual bias cannot be eliminated. We cannot be sure that the SNPs chosen concerning circulating micronutrients will not affect SLE-related outcomes through other causal pathways. Second, there are significant gender differences in SLE, but we cannot stratify the outcome data due to the lack of individual-level data in the summary statistics.

In conclusion, by a two-sample MR analysis and an external validation analysis, our results provided genetic evidence supporting the potential protective role of circulating calcium levels in the risk of SLE. Our findings will provide a crucial scientific basis for dietary intervention in the development and progression of SLE.

## Data availability statement

The original contributions presented in this study are included in this article/Supplementary material, further inquiries can be directed to the corresponding authors.

## Ethics statement

Ethical approval was not required for the study involving humans in accordance with the local legislation and institutional requirements. Written informed consent to participate in this study was not required from the participants or the participants’ legal guardians/next of kin in accordance with the national legislation and the institutional requirements.

## Author contributions

SH: Data curation, Formal analysis, Methodology, Writing – original draft. XW: Data curation, Formal analysis, Methodology, Writing – original draft. FQ: Data curation, Formal analysis, Methodology, Writing – original draft. ZY: Resources, Visualization, Writing – review & editing. CM: Visualization, Writing – review & editing. YK: Validation, Writing – review & editing. CH: Conceptualization, Project administration, Writing – review & editing. JJ: Conceptualization, Project administration, Resources, Writing – review & editing. LY: Conceptualization, Project administration, Resources, Writing – review & editing.

## Footnotes

1
http://www.phenoscanner.medschl.cam.ac.uk/(1-3)
