# Supplementary material for: Assessing causal association of circulating micronutrients and systemic lupus erythematosus susceptibility: a Mendelian randomization study
Source: Front Nutr. 2024 Aug 5;11:1359697. doi: 10.3389/fnut.2024.1359697 (PMC11333035; doi:10.3389/fnut.2024.1359697)
Supplement: Supplementary file 1 [file Data_Sheet_1.docx]

Supplementary Material

# Supplementary Figures and Tables

## Supplementary Figures


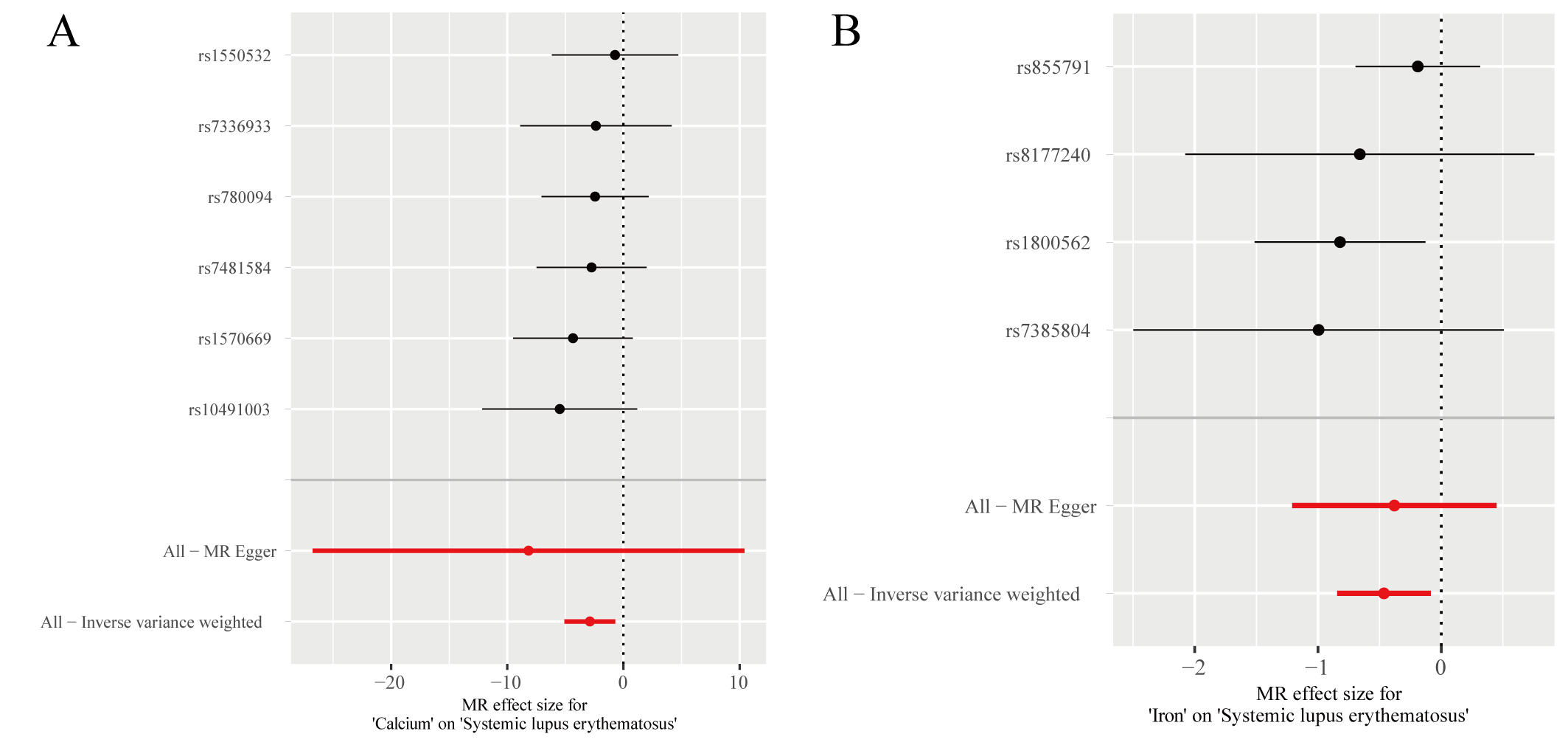


**Supplementary Figure 1.** Forest plots for the impact of individual SNP of circulating calcium (A) and iron (B) on risk of SLE in the European population.


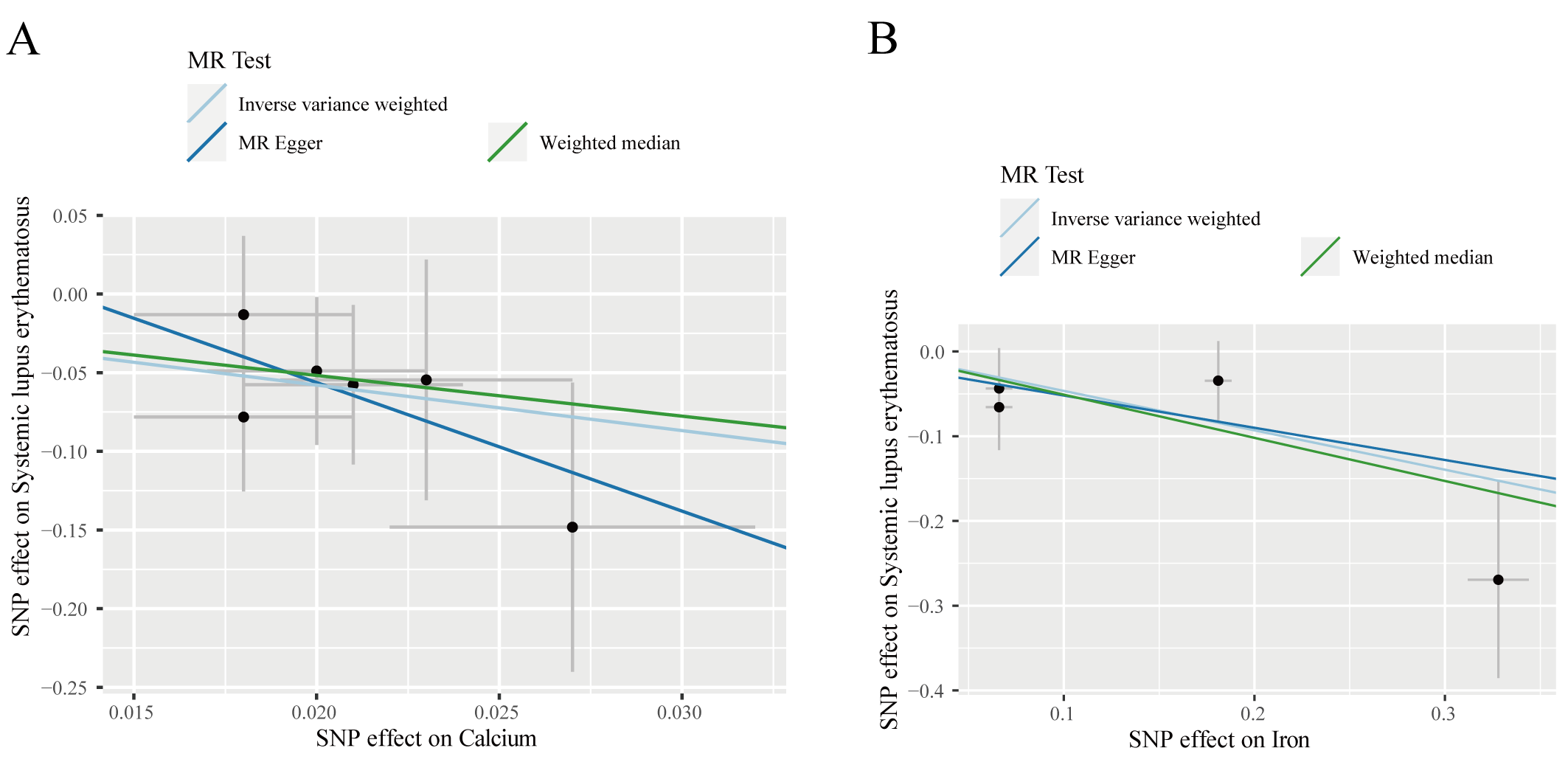


**Supplementary Figure 2.** Scatter plots from genetically predicted circulating calcium (A) and iron (B) on SLE in the European population.


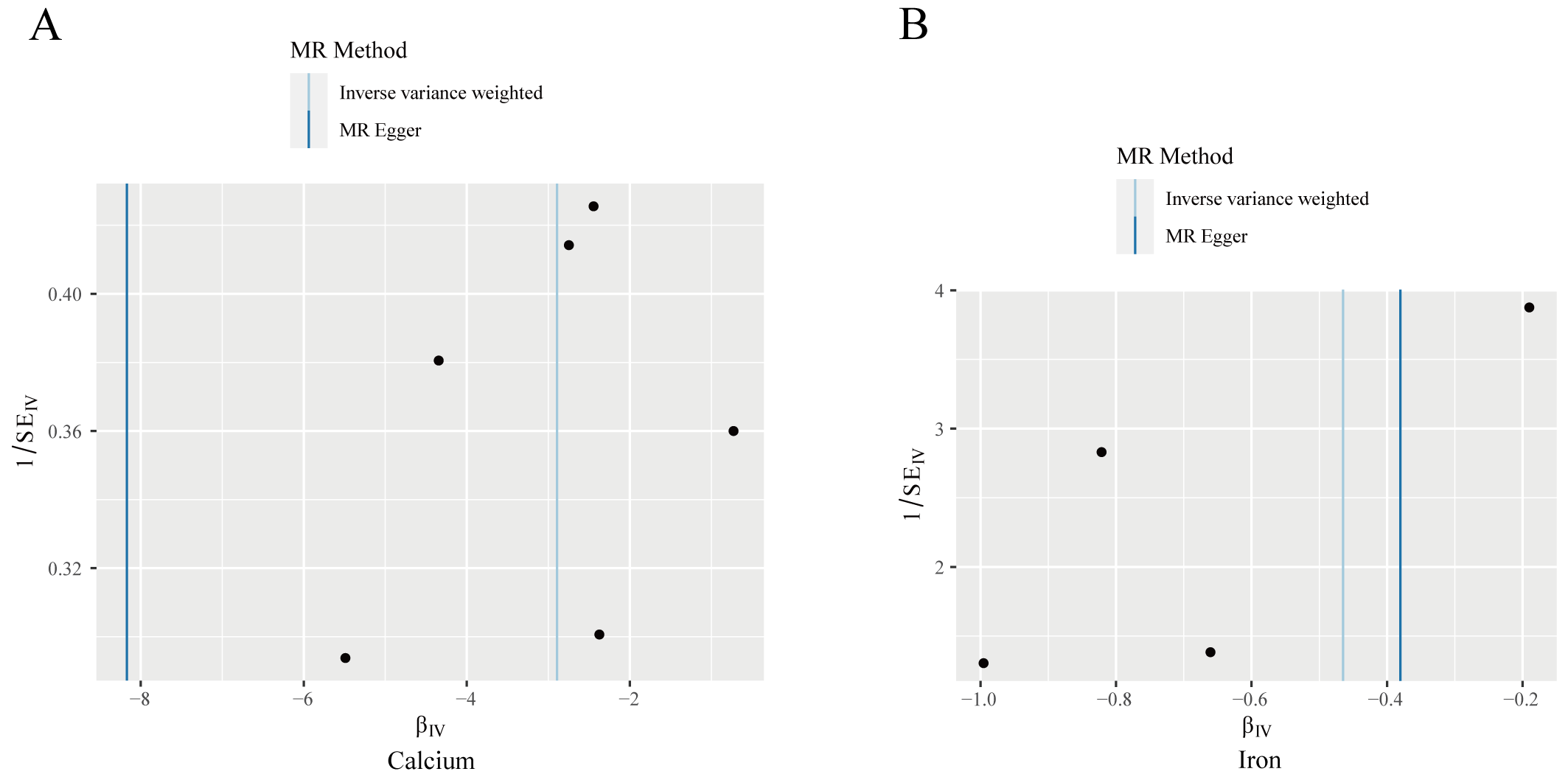


**Supplementary Figure 3.** Funnel plots from genetically predicted circulating calcium (A) and iron (B) on SLE in the European population.


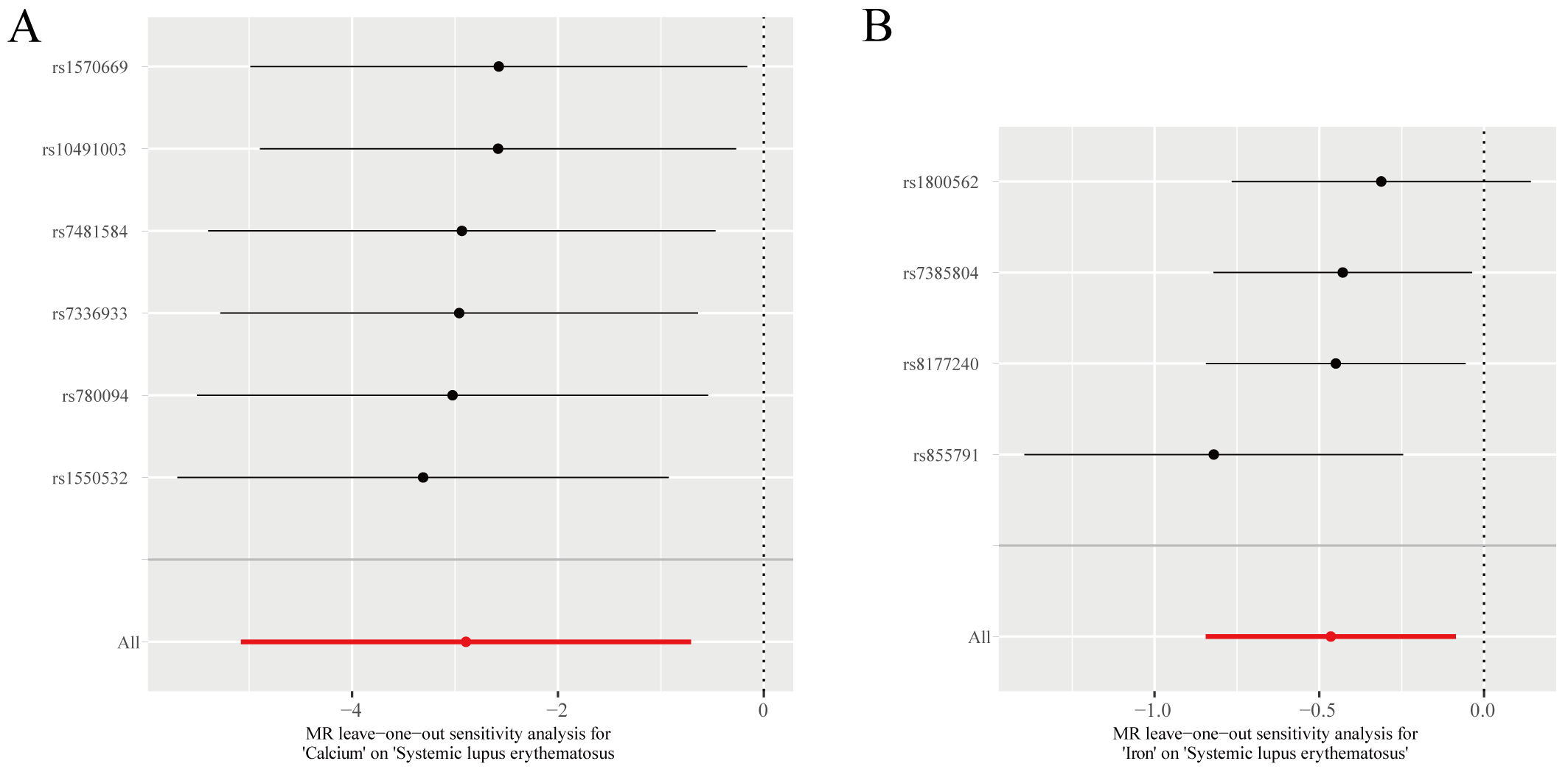


**Supplementary Figure 4.** Leave-one-out plots from genetically predicted circulating calcium (A) and iron (B) on SLE in the European population.

## Supplementary Tables

**Supplementary Table 1** STROBE-MR checklist of recommended items to address in reports of Mendelian randomization studies.

| **Item No.** | **Section** | **Checklist item** | **Page No.** | **Relevant text from manuscript** |
| --- | --- | --- | --- | --- |
| 1 | **TITLE and ABSTRACT** | Indicate Mendelian randomization (MR) as the study’s design in the title and/or the abstract if that is a main purpose of the study |  | Assessing Causal Association of Circulating Micronutrients and Systemic Lupus Erythematosus Susceptibility: A Mendelian Randomization Study |
|  | **INTRODUCTION** |  |  |  |
| 2 | **Background** | Explain the scientific background and rationale for the reported study. What is the exposure? Is a potential causal relationship between exposure and outcome plausible? Justify why MR is a helpful method to address the study question |  | In the Introduction (paragraphs 2-3), we introduce the exposures of interest and the rationale for using MR to explore the causal question. |
| 3 | **Objectives** | State specific objectives clearly, including pre-specified causal hypotheses (if any). State that MR is a method that, under specific assumptions, intends to estimate causal effects |  | Given the multiple advantages of MR in inferring the causal relationship between exposure and outcome, our study utilized a two-sample MR analysis to investigate potential causal relationships between genetically predicted 14 circulating micronutrients (including vitamins and minerals) and the risk of SLE. |
|  | **METHODS** |  |  |  |
| 4 | **Study design and data sources** | Present key elements of the study design early in the article. Consider including a table listing sources of data for all phases of the study. For each data source contributing to the analysis, describe the following: |  |  |
|  | a) | Setting: Describe the study design and the underlying population, if possible. Describe the setting, locations, and relevant dates, including periods of recruitment, exposure, follow-up, and data collection, when available. |  | See Figure 1: A schematic representation of our study design. Abbreviations: SNP, Single Nucleotide Poly-morphism; SLE, Systemic lupus erythematosus; MR, Mendelian Randomization. |
|  | b) | Participants: Give the eligibility criteria, and the sources and methods of selection of participants. Report the sample size, and whether any power or sample size calculations were carried out prior to the main analysis |  | In this research, Single Nucleotide Polymorphisms (SNPs) linked to these 14 circulating micronutrients were designated as instrumental variables (IVs) adhering to the following standards:...; The GWAS summary data (GCST90018917) for SLE were sourced from a recent large-scale GWAS in the IEU OpenGWAS database. This dataset comprises 647 cases of European ancestry (from Finland and the UK) and 482,264 control subjects of European ancestry. |
|  | c) | Describe measurement, quality control and selection of genetic variants |  | (1) The SNP demonstrates significant association with circulating micronutrient (*P* < 5×10^-8^) and lacks linkage disequilibrium (r^2^ < 0.001, KB = 10000) ; (2) The SNP with a minor allele frequency (MAF) of ≥ 5%; (3) The SNP showing no evidence of reverse causality, as determined by the Steiger filtering test; (4) In cases where the SNP is not found in the results dataset, a closely associated SNP (r^2^ > 0.8) is chosen as a proxy in the 1000 Genomes database. If proxy SNP was unavailable, it was excluded from the analysis. (5) The chosen SNP is confirmed to be unassociated with con-founding factors through inspection via the PhenoScanner database (http://www.phenoscanner.medschl.cam.ac.uk/) (*P* < 5×10^-8^，r^2^ = 0.8). Furthermore, we calculated the R^2^ to denote the variance explained by the SNP and the F-statistic to signify potential weak IV bias in MR analysis. The R^2^ was calculated as follows: R^2^ = 2 × Beta^2^ × (1-EAF) × EAF / SD^2^, and the F-statistic was calculated as: F = (Beta)^2^ / (SE)^2^, where Beta is the per allele effect size of the association between each SNP and phenotype, EAF is the effect allele frequency, SE is the standard error, SD is the standard deviation. The IV is deemed strong when the F-statistic ≥ 10 . |
|  | d) | For each exposure, outcome, and other relevant variables, describe methods of assessment and diagnostic criteria for diseases | . |  |
|  | e) | Provide details of ethics committee approval and participant informed consent, if relevant |  |  |
| 5 | **Assumptions** | Explicitly state the three core IV assumptions for the main analysis (relevance, independence and exclusion restriction) as well assumptions for any additional or sensitivity analysis |  | Assumptions introduced in the Introduction and Figure 1. Sensitivity analysis introduced in the statistical analyses section. |
| 6 | **Statistical methods: main analysis** | Describe statistical methods and statistics used |  |  |
|  | a) | Describe how quantitative variables were handled in the analyses (i.e., scale, units, model) |  | Statistical analyses section. |
|  | b) | Describe how genetic variants were handled in the analyses and, if applicable, how their weights were selected |  | Furthermore, we calculated the R^2^ to denote the variance explained by the SNP and the F-statistic to signify potential weak IV bias in MR analysis. |
|  | c) | Describe the MR estimator (e.g. two-stage least squares, Wald ratio) and related statistics. Detail the included covariates and, in case of two-sample MR, whether the same covariate set was used for adjustment in the two samples |  | See Statistical Analyses section. |
|  | d) | Explain how missing data were addressed |  | In cases where the SNP is not found in the results dataset, a closely associated SNP (r^2^ > 0.8) is chosen as a proxy in the 1000 Genomes database. If proxy SNP was unavailable, it was excluded from the analysis. |
|  | e) | If applicable, indicate how multiple testing was addressed |  |  |
| 7 | **Assessment of assumptions** | Describe any methods or prior knowledge used to assess the assumptions or justify their validity |  | Statistical analyses in relation to the verification of MR assumptions are provided in the statistical analyses section. |
| 8 | **Sensitivity analyses and additional analyses** | Describe any sensitivity analyses or additional analyses performed (e.g. comparison of effect estimates from different approaches, independent replication, bias analytic techniques, validation of instruments, simulations) |  | Statistical analyses in relation to the verification of MR assumptions are provided in the statistical analyses section. |
| 9 | **Software and pre-registration** |  |  |  |
|  | a) | Name statistical software and package(s), including version and settings used |  | In this study, *P* < 0.05 was considered statistically significant. All analyses were performed using R software, with the "TwoSampleMR" and "MR-PRESSO" packages facilitating the two-sample MR analysis. |
|  | b) | State whether the study protocol and details were pre-registered (as well as when and where) |  |  |
|  | **RESULTS** |  |  |  |
| 10 | **Descriptive data** |  |  |  |
|  | a) | Report the numbers of individuals at each stage of included studies and reasons for exclusion. Consider use of a flow diagram |  | See Figure 1. |
|  | b) | Report summary statistics for phenotypic exposure(s), outcome(s), and other relevant variables (e.g. means, SDs, proportions) |  | See Table 1 and Supplementary Table 2. |
|  | c) | If the data sources include meta-analyses of previous studies, provide the assessments of heterogeneity across these studies |  |  |
|  | d) | For two-sample MR:  i.  Provide justification of the similarity of the genetic variant-exposure associations between the exposure and outcome samples  ii.  Provide information on the number of individuals who overlap between the exposure and outcome studies |  |  |
| 11 | **Main results** |  |  |  |
|  | a) | Report the associations between genetic variant and exposure, and between genetic variant and outcome, preferably on an interpretable scale |  | See Figure 2 and Supplementary Table 3. |
|  | b) | Report MR estimates of the relationship between exposure and outcome, and the measures of uncertainty from the MR analysis, on an interpretable scale, such as odds ratio or relative risk per SD difference |  | See Table 1 and Supplementary Table 2. |
|  | c) | If relevant, consider translating estimates of relative risk into absolute risk for a meaningful time period |  |  |
|  | d) | Consider plots to visualize results (e.g. forest plot, scatterplot of associations between genetic variants and outcome versus between genetic variants and exposure) |  | See Figure 2 and Supplementary Figure 1-4. Observational results between the exposure and outcome are reported in terms of odds ratio and 95% confidence intervals. MR results are reported in terms of odds ratio and95% confidence intervals. |
| 12 | **Assessment of assumptions** |  |  |  |
|  | a) | Report the assessment of the validity of the assumptions |  | As indicated in Supplementary Table 4, both Cochrane's Q test and the MR-Egger intercept suggest no heterogeneity and horizontal pleiotropy present in our MR analyses (*P* > 0.05). In the MR-PRESSO analyses, the rs1697421 (*P* = 0.01), rs17265703 (*P* = 0.005) and rs1801725 (*P* = 0.005) were identified as outliers. |
|  | b) | Report any additional statistics (e.g., assessments of heterogeneity across genetic variants, such as *I^2^*, Q statistic or E-value) |  | See Supplementary Table 5, We report F statistics and R^2^. |
| 13 | **Sensitivity analyses and additional analyses** |  |  |  |
|  | a) | Report any sensitivity analyses to assess the robustness of the main results to violations of the assumptions |  | See Sensitive analysis section and Supplementary Table 2-5. |
|  | b) | Report results from other sensitivity analyses or additional analyses |  | See Supplementary Table 3 and Results section: Concurrently, the weighted median method also derived similar results regarding the causal relationship between circulating iron level and the risk of SLE (OR: 0.60, 95% CI: 0.39-0.92, *P* = 0.020). |
|  | c) | Report any assessment of direction of causal relationship (e.g., bidirectional MR) |  | See Table 1: MR Steiger test section. |
|  | d) | When relevant, report and compare with estimates from non-MR analyses |  |  |
|  | e) | Consider additional plots to visualize results (e.g., leave-one-out analyses) |  | See leave-one-out analyses (Supplementary Figure 4) and Forest plots for the impact of individual SNP of circulating calcium (A) and iron (B) on risk of SLE in the European population(Supplementary Figure 1). |
|  | **DISCUSSION** |  |  |  |
| 14 | **Key results** | Summarize key results with reference to study objectives |  | Discussion paragraph 1. |
| 15 | **Limitations** | Discuss limitations of the study, taking into account the validity of the IV assumptions, other sources of potential bias, and imprecision. Discuss both direction and magnitude of any potential bias and any efforts to address them |  | Discussion paragraph 5. |
| 16 | **Interpretation** |  |  |  |
|  | a) | Meaning: Give a cautious overall interpretation of results in the context of their limitations and in comparison with other studies |  | Discussion paragraphs 2-3. We present a cautious overall interpretation of results. |
|  | b) | Mechanism: Discuss underlying biological mechanisms that could drive a potential causal relationship between the investigated exposure and the outcome, and whether the gene-environment equivalence assumption is reasonable. Use causal language carefully, clarifying that IV estimates may provide causal effects only under certain assumptions |  | Discussion paragraphs 2-3.We discuss the possible role of circulating calcium and iron on  SLE risk. |
|  | c) | Clinical relevance: Discuss whether the results have clinical or public policy relevance, and to what extent they inform effect sizes of possible interventions |  |  |
| 17 | **Generalizability** | Discuss the generalizability of the study results (a) to other populations, (b) across other exposure periods/timings, and (c) across other levels of exposure |  | However, ZZ W et al. discovered that an overabundance of iron could stimulate the generation of pro-inflammatory cytokines via poly(rC)-binding protein 1 (Pcbp1), consequently leading to the direct induction of autoimmune diseases. Thus, there is still no clear consensus on the mechanism of serum iron's effect on SLE risk. |
|  | **OTHER INFORMATION** |  |  |  |
| 18 | **Funding** | Describe sources of funding and the role of funders in the present study and, if applicable, sources of funding for the databases and original study or studies on which the present study is based |  | Funding requirements completed in accordance with journal guidelines. |
| 19 | **Data and data sharing** | Provide the data used to perform all analyses or report where and how the data can be accessed, and reference these sources in the article. Provide the statistical code needed to reproduce the results in the article, or report whether the code is publicly accessible and if so, where |  | We give access information to all data used in the study and list which software were used. |
| 20 | **Conflicts of Interest** | All authors should declare all potential conflicts of interest |  | The authors declare no conflict of interest. |

**Supplementary Table 2** Summary information for 62 SNPs of 14 circulating micronutrients.

| **Exposure** | **SNPs** | **EA** | **OA** | **EAF** | **Beta** | **SE** | ***P*-value** | **Sample size** |
| --- | --- | --- | --- | --- | --- | --- | --- | --- |
| **Vitamins** |  |  |  |  |  |  |  |  |
| Vitamin A | rs10882272 | C | T | 0.35 | -0.03 | 0.004 | 7.80E-12 | 5006 |
|  | rs1667255 | C | A | 0.31 | 0.03 | 0.004 | 6.35E-14 | 5006 |
| Vitamin B6 | rs4654748 | C | T | 0.50 | -1.45 | 0.280 | 8.30E-18 | 1864 |
| Vitamin B9 | rs1801133 | G | A | 0.67 | 0.11 | 0.008 | 6.65E-53 | 37341 |
|  | rs652197 | C | T | 0.18 | 0.07 | 0.010 | 5.73E-13 | 37341 |
|  | rs76630415 | G | T | 0.21 | -0.04 | 0.007 | 2.40E-08 | 64979 |
| Vitamin B12 | rs1131603 | C | T | 0.06 | 0.19 | 0.017 | 4.30E-28 | 82917 |
|  | rs1141321 | C | T | 0.63 | 0.06 | 0.007 | 1.40E-16 | 82917 |
|  | rs12272669 | A | G | 0.00 | 0.51 | 0.086 | 3.00E-09 | 82917 |
|  | rs1801222 | G | A | 0.59 | 0.11 | 0.007 | 1.10E-52 | 82917 |
|  | rs2270655 | G | C | 0.94 | 0.07 | 0.016 | 3.50E-05 | 82917 |
|  | rs2336573 | T | C | 0.03 | 0.32 | 0.021 | 1.10E-51 | 82917 |
|  | rs34324219 | C | A | 0.88 | 0.21 | 0.012 | 8.80E-71 | 82917 |
|  | rs3742801 | T | C | 0.29 | 0.05 | 0.008 | 5.30E-08 | 82917 |
|  | rs41281112 | C | T | 0.95 | 0.17 | 0.016 | 9.60E-27 | 82917 |
|  | rs602662 | A | G | 0.60 | 0.16 | 0.008 | 4.10E-96 | 82917 |
| Vitamin C | rs10051765 | C | T | 0.34 | 0.04 | 0.007 | 3.64E-09 | 52018 |
|  | rs10136000 | A | G | 0.28 | 0.04 | 0.007 | 1.33E-08 | 52018 |
|  | rs117885456 | A | G | 0.09 | 0.08 | 0.012 | 1.70E-11 | 52018 |
|  | rs13028225 | T | C | 0.86 | 0.10 | 0.009 | 2.38E-30 | 52018 |
|  | rs174547 | C | T | 0.33 | 0.04 | 0.007 | 3.84E-08 | 52018 |
|  | rs2559850 | A | G | 0.60 | 0.06 | 0.006 | 6.30E-20 | 52018 |
|  | rs56738967 | C | G | 0.32 | 0.04 | 0.007 | 7.62E-10 | 52018 |
|  | rs6693447 | T | G | 0.55 | 0.04 | 0.006 | 6.25E-10 | 52018 |
|  | rs9895661 | T | C | 0.82 | 0.06 | 0.008 | 1.05E-14 | 52018 |
|  | ^a^rs10051765 | C | T | 0.34 | 0.04 | 0.007 | 3.64E-09 | 52018 |
| Vitamin D | rs10741657 | A | G | 0.40 | 0.03 | 0.002 | 2.05E-46 | 79366 |
|  | rs10745742 | T | C | 0.40 | 0.02 | 0.002 | 1.88E-14 | 79366 |
|  | rs12785878 | T | G | 0.75 | 0.04 | 0.002 | 3.80E-62 | 79366 |
|  | rs17216707 | T | C | 0.79 | 0.03 | 0.003 | 8.14E-23 | 79366 |
|  | rs3755967 | T | C | 0.28 | -0.09 | 0.002 | 1.00E-200 | 79366 |
|  | rs8018720 | C | G | 0.82 | -0.02 | 0.003 | 4.72E-09 | 79366 |
| Vitamin E | ^c^rs11057830 | A | G | 0.15 | 0.03 | 0.010 | 8.20E-09 | 7781 |
|  | ^c^rs2108622 | T | C | 0.15 | 0.03 | 0.010 | 1.40E-10 | 7781 |
|  | rs964184 | G | C | 0.21 | 0.04 | 0.010 | 7.80E-12 | 7781 |
| **Minerals** |  |  |  |  |  |  |  |  |
| Phosphorus | ^b^rs1697421 | G | A | 0.49 | 0.05 | 0.005 | 1.14E-27 | 16264 |
|  | ^b^rs17265703 | A | G | 0.85 | 0.04 | 0.006 | 4.32E-09 | 16264 |
|  | rs2970818 | T | A | 0.09 | 0.05 | 0.008 | 4.38E-09 | 16264 |
|  | rs9469578 | C | T | 0.92 | 0.06 | 0.009 | 1.11E-11 | 16264 |
|  | rs947583 | T | C | 0.29 | 0.04 | 0.005 | 3.45E-12 | 16264 |
| Calcium | rs10491003 | T | C | 0.09 | 0.03 | 0.005 | 1.60E-06 | 39400 |
|  | rs1550532 | C | G | 0.31 | 0.02 | 0.003 | 4.60E-08 | 39400 |
|  | rs1570669 | G | A | 0.66 | 0.02 | 0.003 | 4.00E-08 | 39400 |
|  | ^b^rs1801725 | T | G | 0.15 | 0.07 | 0.004 | 6.50E-59 | 39400 |
|  | rs7336933 | G | A | 0.15 | 0.02 | 0.004 | 1.60E-07 | 39400 |
|  | rs7481584 | G | A | 0.30 | 0.02 | 0.003 | 9.20E-10 | 39400 |
|  | rs780094 | T | C | 0.42 | 0.02 | 0.003 | 3.70E-11 | 39400 |
| Magnesium | rs11144134 | C | T | 0.08 | 0.01 | 0.001 | 8.20E-15 | 23829 |
|  | rs13146355 | A | G | 0.44 | 0.01 | 0.001 | 6.30E-13 | 23829 |
|  | rs3925584 | T | C | 0.55 | 0.01 | 0.001 | 5.20E-16 | 23829 |
|  | rs4072037 | T | C | 0.54 | 0.01 | 0.001 | 2.00E-36 | 23829 |
|  | rs448378 | A | G | 0.53 | 0.00 | 0.001 | 1.25E-08 | 23829 |
|  | ^a^rs7965584 | A | G | 0.71 | 0.01 | 0.001 | 1.10E-16 | 23829 |
| Copper | rs1175550 | G | A | 0.23 | 0.20 | 0.032 | 5.03E-10 | 2603 |
|  | rs2769264 | G | T | 0.19 | 0.31 | 0.034 | 2.63E-20 | 2603 |
| Iron | rs1800562 | A | G | 0.07 | 0.33 | 0.016 | 2.72E-97 | 48972 |
|  | rs7385804 | T | G | 0.67 | -0.07 | 0.007 | 6.65E-20 | 48972 |
|  | rs8177240 | T | G | 0.67 | -0.07 | 0.007 | 6.65E-20 | 48972 |
|  | rs855791 | G | A | 0.55 | 0.18 | 0.007 | 1.32E-139 | 48972 |
| Zinc | rs1532423 | A | G | 0.43 | 0.18 | 0.026 | 9.00E-12 | 2603 |
|  | rs2120019 | T | C | 0.81 | 0.29 | 0.033 | 1.50E-18 | 2603 |
| Selenium | rs921943 | T | C | 0.29 | 0.25 | 0.023 | 9.40E-28 | 5477 |

Abbreviations: SNPs, single nucleotide polymorphisms; OA, other allele; EA, effect allele; SE, standard error.

^a^rs10051765 and rs7965584 were not available in the outcome dataset, but rs11105470 was found to replace rs 7965584 in the 1000 Genomes database.

^b^rs1697421, rs17265703 and rs1801725 were found to be outlier IV in MR-PRESSO.

^c^rs11057830, rs2108622 were remove because of F < 10 of them, respectively.

**Supplementary Table 3** Two-sample Mendelian randomization estimations showing the effect of 14 circulating micronutrients on the risk of SLE.

| **Exposure** | **SNPs** | **Method** | **OR** | **OR (95%CI)** | | ***P*-value** |
| --- | --- | --- | --- | --- | --- | --- |
|  |  |  |  | **lower** | **upper** |  |
| **Vitamins** |  |  |  |  |  |  |
| Vitamin A | 2 | Inverse variance weighted | 7.00 | 0.25 | 192.85 | 0.250 |
| Vitamin B6 | 1 | Wald ratio | 0.98 | 0.92 | 1.04 | 0.502 |
| Vitamin B9 | 3 | MR Egger | 0.90 | 0.07 | 11.61 | 0.949 |
|  |  | Weighted median | 0.83 | 0.31 | 2.22 | 0.709 |
|  |  | Inverse variance weighted | 0.78 | 0.32 | 1.89 | 0.579 |
| Vitamin B12 | 10 | MR Egger | 0.90 | 0.53 | 1.54 | 0.714 |
|  |  | Weighted median | 1.10 | 0.75 | 1.60 | 0.632 |
|  |  | Inverse variance weighted | 0.96 | 0.71 | 1.31 | 0.818 |
| Vitamin C | 9 | MR Egger | 0.97 | 0.03 | 30.56 | 0.987 |
|  |  | Weighted median | 0.99 | 0.39 | 2.52 | 0.991 |
|  |  | Inverse variance weighted | 1.00 | 0.39 | 2.58 | 0.998 |
| Vitamin D | 6 | MR Egger | 0.59 | 0.11 | 3.23 | 0.578 |
|  |  | Weighted median | 0.67 | 0.24 | 1.86 | 0.439 |
|  |  | Inverse variance weighted | 0.87 | 0.34 | 2.26 | 0.777 |
| Vitamin E | 1 | Wald ratio | 1.09 | 0.06 | 21.00 | 0.952 |
| **Minerals** |  |  |  |  |  |  |
| Phosphorus | 3 | MR Egger | 74.32 | 0.02 | 313412.56 | 0.496 |
|  |  | Weighted median | 0.23 | 0.02 | 2.69 | 0.239 |
|  |  | Inverse variance weighted | 0.16 | 0.02 | 1.66 | 0.125 |
| Calcium | 6 | MR Egger | 0.00 | 0.00 | 33519.52 | 0.438 |
|  |  | Weighted median | 0.08 | 0.00 | 1.18 | 0.066 |
|  |  | Inverse variance weighted | 0.06 | 0.01 | 0.49 | 0.009 |
| Magnesium | 6 | MR Egger | 0.01 | 0.00 | 2062189.34 | 0.657 |
|  |  | Weighted median | 0.06 | 0.00 | 177.28 | 0.486 |
|  |  | Inverse variance weighted | 0.72 | 0.00 | 357.61 | 0.917 |
| Copper | 2 | Inverse variance weighted | 0.98 | 0.65 | 1.46 | 0.906 |
| Iron | 4 | MR Egger | 0.68 | 0.30 | 1.57 | 0.464 |
|  |  | Weighted median | 0.60 | 0.39 | 0.92 | 0.020 |
|  |  | Inverse variance weighted | 0.63 | 0.43 | 0.92 | 0.016 |
| Zinc | 2 | Inverse variance weighted | 1.10 | 0.76 | 1.59 | 0.600 |
| Selenium | 1 | Wald ratio | 1.28 | 0.83 | 1.98 | 0.271 |

Abbreviations: SNPs, single nucleotide polymorphisms; OR, odds ratio; CI, confidence interval.

**Supplementary Table 4** Cochrane’s Q test, MR-Egger intercept and MR-PRESSO Mendelian randomization analyses of 14 circulating micronutrients.

| **Exposure** | **Sample size** | **Cochrane’s Q test^a^**  **(*P*-value)** | **MR-Egger intercept^b^**  **(*P*-value)** | **MR-PRESSO^c^**  **(*P*-value)** |
| --- | --- | --- | --- | --- |
| **Vitamins** |  |  |  |  |
| Vitamin A | 5006 | 0.15 | NA | NA |
| Vitamin B6 | 1864 | NA | NA | NA |
| Vitamin B9 | 37341/64979 | 0.24 | 0.92 | NA |
| Vitamin B12 | 82917 | 0.29 | 0.76 | 0.36 |
| Vitamin C | 52018 | 0.06 | 0.99 | 0.12 |
| Vitamin D | 79366 | 0.67 | 0.62 | 0.64 |
| Vitamin E | 7781 | NA | NA | NA |
| **Minerals** |  |  |  |  |
| Phosphorus | 16264 | 0.34 | 0.38 | 0.68^*^ |
| Calcium | 39400 | 0.87 | 0.61 | 0.93^*^ |
| Magnesium | 23829 | 0.46 | 0.66 | 0.58 |
| Copper | 2603 | 0.19 | NA | NA |
| Iron | 48972 | 0.27 | 0.84 | 0.47 |
| Zinc | 2603 | 0.21 | NA | NA |
| Selenium | 5477 | NA | NA | NA |

^a^The Cochrane’s Q test was used to test heterogeneity (SNP > 1).

^b^The MR-Egger intercept was used to test horizontal pleiotropy (SNP > 1).

^c^The MR-PRESSO method was used to detect the existence of outlier IVs (SNP > 3).

^*^The rs1697421 (P = 0.01), rs17265703 (P = 0.005) and rs1801725 (P = 0.005) were identified as outliers. After the exclusion of outliers, the MR-PRESSO test was re-runed.

**Supplementary Table 5** F-statistics and R^2^ of 14 circulating micronutrients

| **Exposure** | **N** | **NSNP** | **Total R^2^** | **F-statistic** |
| --- | --- | --- | --- | --- |
| **Vitamins** |  |  |  |  |
| Vitamin A | 5006 | 2 | 0.99% | 56.3-56.3 |
| Vitamin B6 | 1864 | 1 | 0.72% | 26.8 |
| Vitamin B9 | 37341 | 3 | 0.29% | 203.1-127.9 |
| Vitamin B12 | 82917 | 10 | 0.56% | 400.0-31.6 |
| Vitamin C | 52018 | 9 | 0.34% | 128.4-26.4 |
| Vitamin D | 79366 | 6 | 1.39% | 1980.3-32.1 |
| Vitamin E | 7781 | 1 | 0.07% | 16.0 |
| **Minerals** |  |  |  |  |
| Phosphorus | 16264 | 3 | 0.20% | 49.0-34.5 |
| Calcium | 39400 | 6 | 0.22% | 297.6-29.2 |
| Magnesium | 23829 | 6 | 0.53% | 121.0-25.0 |
| Copper | 2603 | 2 | 1.50% | 84.7-38.3 |
| Iron | 48972 | 4 | 0.94% | 668.6-88.9 |
| Zinc | 2603 | 2 | 1.78% | 75.6-46.9 |
| Selenium | 5477 | 1 | 0.86% | 114.4 |

R^2^ = 2 × Beta^2^ × (1-EAF) × EAF/SD^2^ , F = (Beta)^2^ / (SE)^2^, SD^2^ = SE^2^ * N where EAF = effect allele frequency, Beta = the effect of each SNP on the exposures, SE = standard error, SD = standard deviation, N = sample size.

**Supplementary Table 6** Demographic characteristics of NHANES III participants by SLE status (N = 20045)

| **Characteristics** | **SLE group**  **(N = 40)** | **Control group**  **(N = 20005)** | ***P*-value**^a^ |
| --- | --- | --- | --- |
| **Age (Mean, SD)** | 52.06 (14.82) | 43.93 (18.46) | <0.01 |
| **BMI (N, %)** |  |  | 0.71 |
| Underweight  <18.5 kg/m^2^ | 2 (5.00%) | 493 (2.50%) |  |
| Normal  18.5–24.9 kg/m^2^ | 14 (35.00%) | 7013 (35.1%) |  |
| Overweight  25–29.9 kg/m^2^ | 16 (40%) | 8046 (40.20%) |  |
| Obese  ≥ 30 kg/m^2^ | 8 (20.00%) | 4443 (22.2%) |  |
| **Gender** |  |  | 0.13 |
| Male | 8 (20.00%) | 9389 (46.90%) |  |
| Female | 32 (80.00%) | 10616 (53.1%) |  |
| **Race** |  |  | 0.33 |
| Mexican American | 12 (30.00%) | 5294 (26.50%) |  |
| Non-Hispanic White | 17 (42.50%) | 8462 (42.30%) |  |
| Non-Hispanic Black | 11 (27.50%) | 5475 (27.4%) |  |
| Other Race-Including Multiracial | 0 (0.00%) | 774 (3.9%) |  |

^a^ Weighted statistics (including percentage) were reported. T-tests, chi-square tests, rank sum tests were conducted by accounting for the complex sampling design.

Abbreviations: NHANES, National Health and Nutritional Examination Survey; SLE, Systemic lupus erythematosus.

**Supplementary Table 7** Differences in 6 circulating micronutrients between the SLE group and Control group among NHANES III participants (N = 20045)

| **Exposure** | **SLE group**  **(N = 40)** | **Control** **group**  **(N = 20005)** | ***P*-value**^a^ |
| --- | --- | --- | --- |
| **Vitamins (Mean, SD)** |  |  |  |
| Vitamin A (ug/dL) | 56.02 (13.72) | 57.80 (13.92) | 0.72 |
| Vitamin C (mg/dL) | 0.70 (0.43) | 0.74 (0.43) | 0.11 |
| Vitamin D (nmol/L) | 60.33 (21.54) | 72.02 (27.91) | <0.01 |
| **Minerals** |  |  |  |
| Calcium (mmol/L) | 2.28 (0.09) | 2.32 (0.10) | 0.01 |
| Iron (umol/L) | 13.79 (4.35) | 16.11 (6.28) | 0.04 |
| Selenium(ug/dL) | 126.04 (12.75) | 124.61 (15.84) | 0.12 |

^a^ Weight statistics (including mean and SD) were reported. T-test was conducted by accounting for complex sampling design.

Abbreviations: NHANES, National Health and Nutritional Examination Survey; SLE, Systemic lupus erythematosus.

**Supplementary Table 8**  Logistic regression analyses of 6 circulating micronutrients for SLE status by adjusting for demographic characteristics of NHANES III participants. (N = 20045)

| **Exposure** | **Univariate risk factor** | | **Multivariate risk factors** | |
| --- | --- | --- | --- | --- |
|  | **OR (95% CI)** | ***P*-value** | **OR (95% CI)** | ***P*-value** |
| **Vitamins** |  |  |  |  |
| Vitamin A (ug/dL) | 1.01(1.00, 1.03) | 0.16 |  |  |
| Vitamin C (mg/dL) | 0.41(0.17, 0.97) | 0.04 |  |  |
| Vitamin D (nmol/L) | 0.98(0.96,0.99) | <0.01 | 0.98(0.97, 1.00) | 0.01 |
| **Minerals** |  |  |  |  |
| Calcium (mmol/L) | 0.02(0.00, 0.43) | 0.01 | 0.03(0.00, 0.58) | 0.02 |
| Iron (umol/L) | 0.96(0.91, 1.02) | 0.15 |  |  |
| Selenium (ug/dL) | 1.01(1.00, 1.02) | 0.04 | 1.01(1.00, 1.02) | 0.01 |

Logistic regression models were adjusted for age, gender, race, and BMI.

Abbreviations: NHANES, National Health and Nutritional Examination Survey; SLE, Systemic lupus erythematosus.
